# Multifocal Noncontiguous Spinal Tuberculosis

**DOI:** 10.4269/ajtmh.23-0060

**Published:** 2023-08-28

**Authors:** Shutao Gao, Yukun Hu, Weibin Sheng

**Affiliations:** Department of Spine Surgery, Xinjiang Medical University Affiliated First Hospital, Urumqi, China

**Figure 1.**
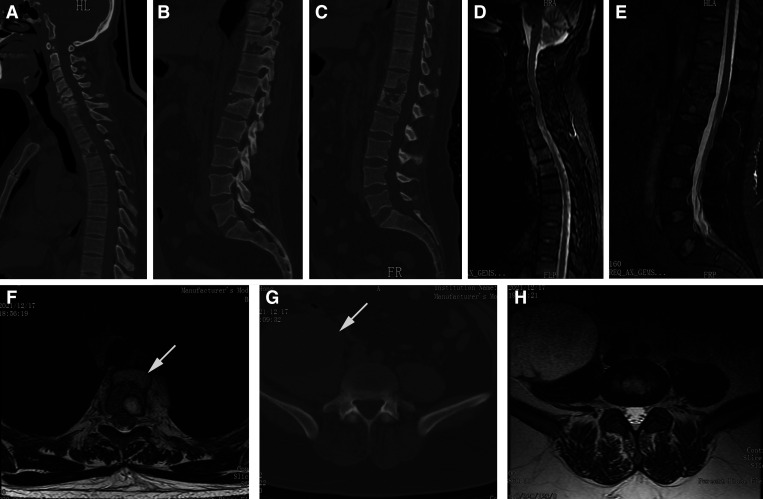
Preoperative CT and MRI scans. (**A–C**) Sagittal CT images indicate bony destruction in the C5, C6, T3, T10, T12, and L1 vertebrae. (**D** and **E**) Preoperative sagittal MRI indicates high signal intensity in multiple vertebrae and a fusiform paraspinal abscess in front of the T1–T4 vertebrae. (**F**) Axial MRI scan at the T4 vertebra level indicate a paravertebral abscess (white arrow). (**G** and **H**) Axial CT and MRI scans at the L4/5 intervertebral level indicate a massive psoas abscess (white arrow). CT = computed tomography; MRI = magnetic resonance imaging.

A 43-year-old man presented to the outpatient department after 2 months of back pain. He denied any medical history of tuberculosis (TB). Physical examination showed no neurological deficit. Laboratory tests indicated a normal white cell count, an increased erythrocyte sedimentation rate (ESR; 40 mm/hour), and an elevated level of C-reactive protein (CRP; 81 mg/L). In addition, the interferon-γ release assay (T-spot test) was positive. Pulmonary computed tomography (CT) did not show a TB focus. Sagittal CT scans indicated bony destruction in the C5, C6, T3, T10, T12, and L1 vertebrae (Figure 1A–C). Magnetic resonance imaging (MRI) examinations suggested abnormal signal intensity in multiple vertebrae (Figure 1D and E) and a fusiform paraspinal abscess at the T1–T4 vertebrae (Figure 1D and F). Axial CT and MRI scans at the L4/5 intervertebral level indicated a massive psoas abscess (Figure 1G and H).

**Figure 2.**
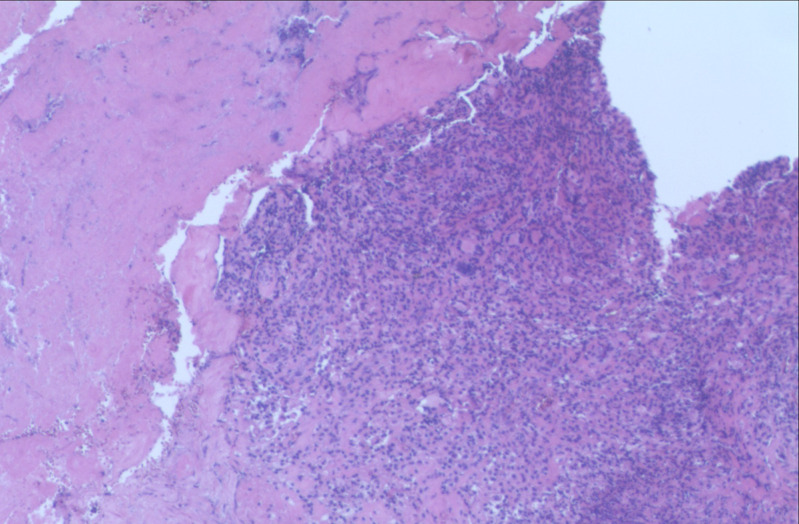
Histopathological examination showed granulomata with multinucleated giant cells (40×).

**Figure 3.**
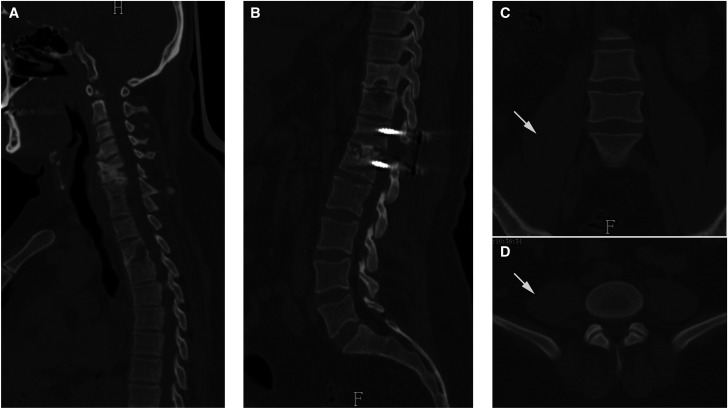
CT images at 12-month follow-up. (**A** and **B**) Sagittal CT images suggest the bony destruction improved in the C5, C6, T3, T10, T12, and L1 vertebrae, and bony fusion was achieved between the T12 and L1 vertebrae. (**C** and **D**) Coronal and axial CT scans indicate that the psoas abscess was cured (white arrows). CT = computed tomography.

With a high suspicion of spinal TB, anti-TB chemotherapy (isoniazid, rifampicin, ethambutol, and pyrazinamide) was prescribed. In the following 2 weeks, the patient’s back pain did not resolve. Given the intolerable back pain and large psoas abscess, surgical treatment was recommended. The patient underwent one-stage anterior approach debridement of the psoas abscess followed by posterior debridement, bone grafting, and internal fixation for the T12/L1 vertebrae. Histopathological examination showed granulomata with multinucleated giant cells, supporting the diagnosis of spinal TB (Figure 2), and anti-TB chemotherapy was continued. The patient’s symptoms significantly improved postoperatively. At 12-month follow-up, the ESR and CRP values had decreased to normal levels. Computed tomography images suggested that the bony destruction had significantly improved (Figure 3A and B) and the psoas abscess was cured (Figure 3C and D).

Despite Garg et al.^1^ reporting that multifocal noncontiguous spinal TB accounted for 2.8% of overall cases, disease involving the whole spine is rarely reported.^2^ If not properly treated, affected individuals may suffer from progressive kyphotic deformity, neurological deficits, and disability.^3^ Treatments for spinal TB include medication and surgery. Anti-TB chemotherapy is the mainstay of treatment, although for patients with a progressive neurological deficit, large abscess, and intolerable pain, surgical treatment becomes inevitable.^4^ After surgery, regular and long-term anti-TB chemotherapy and close follow-up are encouraged.
